# Remifentanil vs. dexmedetomidine for cardiac surgery patients with noninvasive ventilation intolerance: a multicenter randomized controlled trial

**DOI:** 10.1186/s40560-024-00750-2

**Published:** 2024-09-18

**Authors:** Guang-wei Hao, Jia-qing Wu, Shen-ji Yu, Kai Liu, Yan Xue, Qian Gong, Rong-cheng Xie, Guo-guang Ma, Ying Su, Jun-yi Hou, Yi-jie zhang, Wen-jun Liu, Wei Li, Guo-wei Tu, Zhe Luo

**Affiliations:** 1 Department of Cardiac Intensive Care Center, Zhongshan Hospital, Fudan University, Shanghai, 200032 China; 2 Department of Nursing, Zhongshan Hospital, Fudan University, Shanghai, 200032 China; 3 Department of Critical Care Medicine, Zhongshan Hospital, Fudan University, Shanghai, 200032 China; 4 Department of Cardiovascular Surgery, The First Affiliated Hospital of Anhui Medical University, Hefei, 230032 Anhui China; 5 Department of Critical Care Medicine, Xiamen Branch, Zhongshan Hospital, Fudan University, Xiamen, 361015 Fujian China; 6 Department of Intensive Care Unit, The People’s Hospital of Fujian Traditional Medical University, Fuzhou, 350004 Fujian China; 7 Department of Critical Care Medicine, Shanghai Xuhui Central Hospital, Zhongshan Xuhui Hospital, Fudan University, Shanghai, 200020 China; 8 Shanghai Key Lab of Pulmonary Inflammation and Injury, Zhongshan Hospital, Fudan University, Shanghai, 200032 China

**Keywords:** Remifentanil, Dexmedetomidine, Non-invasive ventilation intolerance, Cardiac surgery

## Abstract

**Background:**

The optimal sedative regime for noninvasive ventilation (NIV) intolerance remains uncertain. The present study aimed to assess the efficacy and safety of remifentanil (REM) compared to dexmedetomidine (DEX) in cardiac surgery patients with moderate-to-severe intolerance to NIV.

**Methods:**

In this multicenter, prospective, single-blind, randomized controlled study, adult cardiac surgery patients with moderate-to-severe intolerance to NIV were enrolled and randomly assigned to be treated with either REM or DEX for sedation. The status of NIV intolerance was evaluated using a four-point NIV intolerance score at different timepoints within a 72-h period. The primary outcome was the mitigation rate of NIV intolerance following sedation.

**Results:**

A total of 179 patients were enrolled, with 89 assigned to the REM group and 90 to the DEX group. Baseline characteristics were comparable between the two groups, including NIV intolerance score [3, interquartile range (IQR) 3–3 vs. 3, IQR 3–4, *p* = 0.180]. The chi-squared test showed that mitigation rate, defined as the proportion of patients who were relieved from their initial intolerance status, was not significant at most timepoints, except for the 15-min timepoint (42% vs. 20%, *p* = 0.002). However, after considering the time factor, generalized estimating equations showed that the difference was statistically significant, and REM outperformed DEX (odds ratio = 3.31, 95% confidence interval: 1.35–8.12, *p* = 0.009). Adverse effects, which were not reported in the REM group, were encountered by nine patients in the DEX group, with three instances of bradycardia and six cases of severe hypotension. Secondary outcomes, including NIV failure (5.6% vs. 7.8%, *p* = 0.564), tracheostomy (1.12% vs. 0%, *p* = 0.313), ICU LOS (7.7 days, IQR 5.8–12 days vs. 7.0 days, IQR 5–10.6 days, *p* = 0.219), and in-hospital mortality (1.12% vs. 2.22%, *p* = 0.567), demonstrated comparability between the two groups.

**Conclusions:**

In summary, our study demonstrated no significant difference between REM and DEX in the percentage of patients who achieved mitigation among cardiac surgery patients with moderate-to-severe NIV intolerance. However, after considering the time factor, REM was significantly superior to DEX.

*Trial registration* ClinicalTrials.gov (NCT04734418), registered on January 22, 2021. URL of the trial registry record: https://register.clinicaltrials.gov/prs/app/action/SelectProtocol?sid=S000AM4S&selectaction=Edit&uid=U00038YX&ts=3&cx=eqn1z0.

**Supplementary Information:**

The online version contains supplementary material available at 10.1186/s40560-024-00750-2.

## Introduction

Over the past two decades, noninvasive ventilation (NIV) has undergone a remarkable expansion in its indications, availability, and achievable outcomes [1–5]. At the same time, due to both cardiogenic pulmonary edema and etiology of the lung itself, acute respiratory failure has become common in cardiac surgical intensive care units (CSICUs) [6, 7]. This surge in NIV demand underscores its significance in managing these patients. Furthermore, the implementation of sequential NIV among high-risk cardiac surgery patients has the potential to facilitate weaning from invasive mechanical ventilation to spontaneous breathing [8, 9]. However, NIV intolerance might impose limitations on its utilization. Although notable efforts have been dedicated to enhancing patient comfort during NIV over the past decade and substantial progress has been achieved [10], intolerance to NIV, one of the primary causes contributing to its failure [11, 12], remains a hurdle that curtails its broader adoption.

Previous studies have demonstrated that sedation use can enhance NIV efficacy in patients experiencing NIV intolerance [13–15]. However, the optimal sedative regimen remains uncertain [16, 17]. Dexmedetomidine (DEX) is a highly selective alpha-2 adrenergic receptor agonist with sedative, analgesic, and opioid-sparing effects [18], while remifentanil (REM), an ultra-short-acting opioid with *μ* selectivity, is usually used as a sedative in the ICU setting [19–21]. Besides, both REM and DEX have been used in NIV intolerance [14, 15]. DEX used to be favored as a sedative in various clinical contexts [18, 22], including the CSICU [23, 24]. However, the prevalence of adverse effects associated with DEX, such as bradycardia and hypotension [25, 26], renders it less suitable for numerous cardiac surgery patients who often require vasoactive medications. On the other hand, opioids have been reported to have a protective function in heart tissue and are often used for treating various cardiovascular diseases, such as congestive heart failure, ischemic heart disease, and arrhythmia [27]. As an ultra-short-acting opioid, REM has been reported to be safe and effective in non-cardiac surgery patients with NIV intolerance by two prospective, uncontrolled clinical investigations [28, 29]. Although bradycardia and hypotension have also been reported with REM use, they occurred in the operation room, during anesthesia, and at a very high dosage [30]. In ICU patients, REM was generally associated with an acceptable degree of hemodynamic stability [21, 31, 32]. In addition, the elimination half-life of REM is < 10 min [33, 34], which meant that this side effect could quickly be offset after discontinuation of REM use. Based on these characteristics, REM might be a very attractive option for addressing NIV intolerance in cardiac surgery patients.

Therefore, the present multicenter randomized controlled trial was conducted to further validate and compare the efficacy and safety of REM and DEX in cardiac surgery patients with NIV intolerance. A detailed study protocol was also documented and published [35].

## Methods

### Study design

The present multicenter, prospective, single-blind (with only the enrolled patient blinded to the experimental conditions), randomized controlled trial (registered under ClinicalTrials.gov identifier: NCT04734418) aimed to assess the effectiveness and safety of both REM and DEX in patients undergoing cardiac surgery who developed moderate-to-severe NIV intolerance. The Ethical Committee of Zhongshan Hospital, Fudan University approved the study (No. B2020-374R). Ethical approval for the study protocol, along with any subsequent amendments, was secured from the Ethics Committee at each participating center. The study rigorously adhered to the principles outlined in the Declaration of Helsinki. Written informed consent was obtained from the relatives of all participating patients prior to the commencement of any study-related procedures.

### Population

The study inclusion criteria were as follows: (1) adult cardiac surgery patients; (2) patients who received NIV; and (3) development of moderate-to-severe intolerance. Patients were screened upon NIV initiation and were subsequently enrolled if moderate-to-severe intolerance developed. As detailed in a previous study, NIV tolerance within the study was established using a four-point NIS [36], which was validated by other investigations [37, 38]. The bedside nurse assessed the NIS in the present study. A detailed description of NIS is shown in Table 1. Briefly, a score of 1 indicated a tolerant patient who felt comfortable and relaxed with NIV; a score of 2 indicated a mildly intolerant patient who felt some degree of discomfort and occasionally grabbed at the NIV mask; a score of 3 indicated a moderate intolerant patient who felt discomfort with the NIV mask most of the time and frequently grabbed at the mask (sometimes pulled it off); and a score of 4 indicated a severe intolerant patient who was agitated and/or unable to leave the NIV mask in place. The exclusion criteria were defined as follows: (1) visual analogue scale (VAS) score of ≥ 4; (2) history of allergy to any study drug constituent; (3) expectoration difficulty; (4) severe liver dysfunction (Child–Turcotte–Pugh level C]; (5) renal failure (patients undergoing renal replacement therapy (RRT); (6) preoperative left ventricular ejection fraction (LVEF) of < 30%; (7) mental illness or cognitive impairment; (8) administration of DEX within 8 h or REM within 2 h prior to study commencement; (9) pregnancy or lactation; and (10) delirium prior to the initiation of recruitment.

**Table 1:** Noninvasive ventilation intolerance score

Score	Classification	Description
1	Tolerance	Comfort and relaxation
2	Mild intolerance	Some degree of discomfort and occasionally grabbed at the NIV mask
3	Moderate intolerance	Discomfort with the NIV mask most of the time and frequently grabbed at it (sometimes pulled it off)
4	Severe intolerance	Agitated and/or were unable to maintain the NIV mask in position

### Randomization and sedative interventions

Patients demonstrating moderate or severe NIV intolerance were randomly allocated to either the REM or the DEX group at a 1:1 ratio. Block randomization was carried out with a block size of 4. The random allocation sequence was created using SAS statistical software, version 9.4 (SAS Institute, Cary, NC, USA). Sequentially numbered sealed envelopes were used for randomization. The trial patients were blinded to the treatment assignments. REM (Ruijie, 1 mg, Yichang Humanwell Pharmaceutical Co., Ltd.) was administered intravenously, commencing with an initial dosage of 0.05 μg/kg/min, while DEX (dexmedetomidine hydrochloride injection, 0.2 mg, Yangtze River Pharmaceutical Group) was initiated at a dose of 0.5 μg/kg/h. The infusion rate was adjusted at increments of 0.01 µg/kg/min for REM and 0.1 µg/kg/h for DEX, with the aim of achieving a targeted NIS of ≤ 2. Notably, the upper limits for the REM and DEX infusion rates were set at 0.12 μg/kg/min and 1.0 μg/kg/h, respectively. Midazolam was administered as required in cases where NIV intolerance persisted even after reaching the maximum doses of REM and DEX.

In the present study, analgesic was routinely provided after surgery via a local anesthetic infiltration of ropivacaine, with the catheter inserted at the median sternotomy incision location. In addition, a patient-controlled analgesic pump with sufentanil (1 μg/mL) was provided as needed, with the background infusion rate of 0 mL/h. If the patients felt pain or had a VAS score of 4, a bolus of 3–4 mL was administered.

### NIV management

In the present study, NIV was executed utilizing a facial mask (ZS-MZ-A Face Mask; Shanghai Zhongshan Medical Technology, Shanghai, China) in conjunction with an ICU ventilator equipped with a heated humidifier.

The criteria for NIV initiation were as follows: (1) early extubation with sequential NIV for patients who failed the spontaneous breathing trial (SBT) but met the criteria for weaning from invasive mechanical ventilation [8]. The criteria for SBT failure included respiratory rate of > 30 breaths/min or rapid shallow breathing index (respiratory rate/tidal volume) of > 105 breaths/min/L, PaO_2_/FiO_2_ < 200 mmHg, SpO_2_ < 90%, 20% increase or decrease from the baseline heart rate or blood pressure, use of accessory muscles, paradoxical abdominal movement, and substantial agitation, anxiety, or diaphoresis; (2) sequential NIV for high-risk patients who passed the SBT: body mass index (BMI) of > 30, LVEF of < 40%, and failure of previous extubation [35]; and (3) new onset of acute respiratory failure, with patients meeting at least one of the following criteria: PaO_2_/FiO_2_ of < 200 mmHg, respiratory rate of > 25 breaths/min for at least 2 h, and signs of increased work of breathing, including the use of accessory respiratory muscles and/or paradoxical respiration [36, 39]. In this study, after enrollment, the clinicians would categorize the reasons for NIV into cardiogenic and noncardiogenic, according to the patient’s clinical manifestations, laboratory tests, and bedside examinations, such as chest X-ray, point of care ultrasound, and echocardiography. Briefly, if evidence of cardiogenic pulmonary edema was found, the reason for NIV would be cardiogenic, otherwise the reason would be non-cardiogenic.

The initial NIV settings based on the patient status and NIV goals in the present study were as follows: level of pressure support (PS): 5–15 cm H_2_O; positive end-expiratory pressure (PEEP): 4–10 cm H_2_O; inspiratory trigger: as high as possible while avoiding auto-triggering; expiratory trigger: 25–30%; and FiO_2_: set to the lowest level necessary to achieve the SpO_2_ target. The NIV targets included the following: tidal volume (Vt): 6–8 mL/kg predicted body weight; respiratory rate ≤ 25 breaths/min; PaO_2_/FiO_2_ ≥ 200 mmHg; and SpO_2_: 95–98%. The VAS scores in both groups were regularly collected by bedside nurses. Analgesic drugs were administered as needed to maintain a target pain control level of 0–2. All patients received close monitoring by intensivists and respiratory therapists for intermittent or continuous NIV requirements.

The PS level was titrated to 5 cmH_2_O for more than 2 h, and the patients were weaned by removing the facial mask and breathing spontaneously with oxygen supplementation. The decision to reintroduce NIV was made based on the patient’s clinical condition if the following were observed: (I) SPO_2_ < 94%; (II) RR ≥ 25 breaths/min; and/or (III) signs of increased work of breathing, use of accessory respiratory muscles, and/or paradoxical abdominal movement. NIV success was defined as the absence of ventilator support for a continuous period exceeding 48 h.

The intubation criteria included: (1) tachypnea with a respiratory rate of > 35 breaths/min and the use of accessory muscles; (2) refractory hypoxemia defined as either PaO_2_ of < 50 mmHg or PaO_2_/FiO_2_ of < 100 mmHg; (3) respiratory acidosis, indicated by a pH level of < 7.30 and a PaCO_2_ level of > 50 mmHg; (4) development of conditions necessitating airway protection, such as coma or seizures; and (5) severe hemodynamic instability and life-threatening arrhythmias.

### Data collection

Baseline, demographic, laboratory, and echocardiographic variables were systematically gathered from the electronic medical record system. NIV-related parameters, including the level of PS, PEEP, FiO_2_, Vt, NIS, VAS score, and medication dosage, were collected by bedside nurses at baseline and at the following timepoints: 15 min, 1 h, 3 h, 6 h, and 12 h after the initiation of sedation. In addition, these parameters were recorded every 12 h thereafter until NIV was either discontinued or until 72 h elapsed from the commencement of sedation. All data obtained in the study were entered and securely stored within an Electronic Data Capture System (Happy Life Tech. Co., Ltd., Beijing, China).

### Definitions

In this study, the NIV status was categorized into one of four states: failure, intolerance, tolerance, and liberation. NIV failure was diagnosed when patients required reintubation or faced mortality within 72 h. NIV intolerance was noted when patients had an NIS of 3 or 4. NIV tolerance was established if patients exhibited an NIS of 1 or 2. NIV liberation was concluded when a significant improvement in the patient's condition was achieved and they no longer required NIV support.

In this study, to compare the effect of sedatives, NIV mitigation, which was defined by NIV tolerance or liberation, was adopted. Specifically, patients initially presenting with moderate or severe NIV intolerance (NIS ≥ 3) were considered to have achieved mitigation if their NIS score decreased to ≤ 2 or if they were successfully weaned from NIV. The rational for this definition was according to the actual situation of clinical practice and previous studies [11, 38, 40–43].

In this study, delirium was assessed by the widely used Confusion Assessment Method for the ICU (CAM–ICU) [44–46].

### Study outcomes

The primary study outcome was the percentage of patients who achieved mitigation following sedation with either REM or DEX. In-hospital mortality, ICU length of stay (LOS), duration of NIV support, intubation rate, tracheostomy rate, incidence of delirium, and hemodynamic changes served as the secondary outcomes.

Safety was evaluated using vigilant monitoring of adverse events (AEs) and serious AEs graded in accordance with the Common Terminology Criteria for Adverse Events, version 5.0. AEs and treatment-emergent AEs were systematically categorized using the Medical Dictionary for Regulatory Activities, version 24.1.

### Sample size determination

The pilot study results revealed that the mitigation rate of NIV intolerance up to 1 h, 3 h, 6 h, 12 h, 24 h, 36 h, 48 h, 60 h, and 72 h after REM sedation ranged from 84 to 88%, while the DEX range was 68% to 81% [36]. The mitigation rate of the two groups was close to a maximum after 3 h of treatment and then tended to stabilize. Considering that the faster the onset of sedation, the quicker the mitigation of NIV intolerance and the higher the probability of mitigation achievement, the mitigation rate up to 3 h was considered to be of clinical importance based on the investigator consensus. As a result, the mitigation rate of NIV intolerance was set to be 88% in the REM group and 70% in the DEX group. For a significance level of 5% (*α* = 0.05) and a power of 80% (*β* = 0.2), the analysis showed that 80 subjects per group would be sufficient to detect a difference between the two groups. Assuming a 10% dropout rate, the final sample size was set at 89 patients per group.

### Statistical analysis

All statistical analyses were conducted using SAS statistical software, version 9.4 (SAS Institute, Cary, NC, USA). Data were presented as either mean (standard deviation, SD) or median [25–75% interquartile range (IQR)] for continuous variables and as count (%) for categorical variables. The normality of distribution for continuous variables was assessed through the Kolmogorov–Smirnov test. Either the Student's *t* test or the Mann–Whitney *U* test was employed to compare continuous variables between the two groups depending on the fulfillment of statistical assumptions. Categorical variables were compared between the two groups using the chi-square test or Fisher’s exact test. Considering that the two drugs may have different patterns of action and that efficacy may have a cumulative effect over time, the generalized estimating equations (GEE) method was used to evaluate the dynamic changes in the therapeutic effect over time within the time frame and to evaluate the difference in overall efficacy between the two groups. Specifically, mitigation rate served as the dependent variable in this model, while the different study treatment groups were the independent variables. To account for within-subject correlations over time, an autoregressive correlation structure was specified for the working correlation matrix. The binary outcome of NIV intolerance mitigation was modeled using the logit link function. The analysis was performed using the GENMOD procedure within the SAS software package. Statistical significance was defined as a *p* value of < 0.05.

## Results

### Perioperative characteristics

**Fig. 1:**
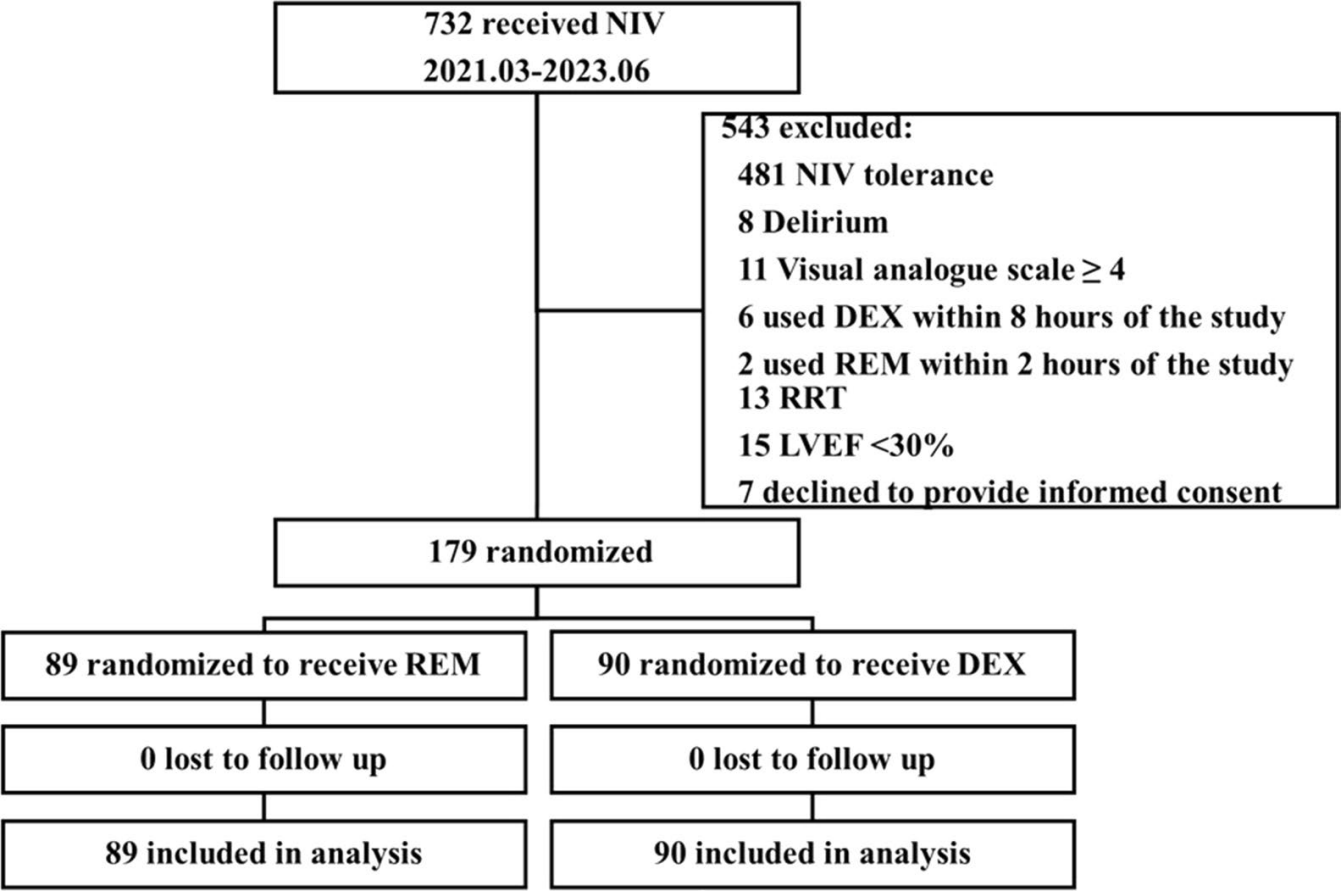
Flow diagram for the study. *NIS* noninvasive ventilation intolerance score, *NIV* noninvasive ventilation, *DEX* dexmedetomidine, *REM* remifentanil, *RRT* renal replacement therapy, *LVEF* left ventricular ejection fraction

The study was conducted at three centers in China and was initiated on March 8, 2021. A total of 732 cardiac surgery patients who received NIV were initially screened and a total of 179 patients were enrolled between March 2021 and June 2023. Among the 732 patients screened, the reasons for exclusion were as follows: 481 patients tolerated NIV, eight developed delirium before sedation, 11 had a VAS score of ≥ 4, eight used the study drug outside of the specified time frame, 13 underwent RRT, 15 had an LVEF of < 30%, and seven declined to provide informed consent. Of the 179 enrolled patients, 89 were assigned to the REM group and 90 were assigned to the DEX group. No patient was lost to the follow-up. The flowchart for the study was shown in Fig. 1.

The perioperative characteristics of the enrolled patients were summarized in Table 2. The median age of the patients was 63 years, and the median BMI stood at 24.8 kg/m^2^. Notably, there were no significant differences in baseline characteristics between the two groups, including age, gender, BMI, history of smoking or alcohol use, comorbidities, and NYHA classification (*p* > 0.05). Moreover, surgery-related variables, such as surgery type, surgical duration, percentage of cardiopulmonary bypass (CPB), CPB duration, and aortic cross-clamp duration, were all found to be comparable between the two groups. In addition, both the Acute Physiology and Chronic Health Evaluation II score and the European System for Cardiac Operative Risk Evaluation demonstrated similar values for both groups.

**Table 2:** Perioperative characteristics of patients with NIV intolerance

Variables	Overall (*n* = 179)	REM (*n* = 89)	DEX (*n* = 90)	*p* value
Age, years	63 (56, 70)	64 (58, 70)	63 (55, 70)	0.732
Gender				0.487
Male, *n* (%)	123 (68.72)	59 (66.29)	64 (71.11)	
Female, *n* (%)	56 (31.28)	30 (33.71)	26 (28.89)	
Height, cm	166.0 (160.0, 172.0)	165 (159.0, 172.0)	166 (161.0, 172.0)	0.605
Weight, kg	70.0 (60.0, 75.5)	68.0 (58.0, 75.0)	70.0 (60.0, 79.5)	0.218
BMI, kg/m^2^	24.80 (22.37, 27.63)	24.06 (22.37, 27.18)	25.12 (22.37, 28.57)	0.195
Smoking history, *n* (%)	38 (21.23)	17 (19.10)	21 (23.33)	0.489
Alcohol history, *n* (%)	25 (13.97)	12 (13.48)	13 (14.44)	0.853
Comorbidities				
Hypertension, *n* (%)	99 (55.31)	48 (53.93)	51 (56.67)	0.713
Diabetes, *n* (%)	27 (15.08)	15 (16.85)	12 (13.33)	0.511
Others, *n* (%)	49 (27.37)	24 (26.97)	25 (27.78)	0.903
NYHA classification				0.886
I, *n* (%)	3 (1.68)	2 (2.27)	1 (1.11)	
II, *n* (%)	52 (29.05)	25 (28.41)	27 (30.00)	
III, *n* (%)	107 (59.78)	54 (61.36)	53 (58.89)	
IV, *n* (%)	16 (8.94)	7 (7.95)	9 (10.00)	
Type of surgery				0.625
Valve only, *n* (%)	76 (42.46)	38 (42.70)	38 (42.22)	
CABG, *n* (%)	25 (13.97)	16 (17.98)	9 (10.00)	
Valve and CABG, *n* (%)	19 (10.61)	9 (10.11)	10 (11.11)	
Great vessel, *n* (%)	46 (25.70)	19 (21.35)	27 (30.00)	
Congenital heart disease, *n* (%)	2 (1.12)	1 (1.12)	1 (1.11)	
Others	11 (6.15)	6 (6.74)	5 (5.56)	
Surgery duration, h	5 (4, 6)	5(4, 6)	5(4, 6)	0.474
CPB, *n* (%)	159 (88.8)	77 (86.5)	82 (91.1)	0.329
CPB duration, min	144 (110, 180)	141 (96, 177)	147 (125, 180)	0.291
Aortic cross-clamp duration, min	77 (56, 99)	81 (51, 97)	75 (58, 100)	0.919
APACHE II	9 (7, 13)	8 (6, 13)	10 (7, 13)	0.334
EuroSCORE	5 (3, 7)	5 (2, 7)	5 (3, 7)	0.387

Continuous data were presented as mean (SD) or median (IQR). Categorical data are presented as counts (%)

*NIV* noninvasive ventilation, *BMI* body mass index, *NYHA* New York Heart Association, *CABG* coronary artery bypass graft, *CPB* cardiopulmonary bypass, *APACHE* Acute Physiology and Chronic Health Evaluation, *EuroSCORE* European system for cardiac operative risk evaluation

### Baseline characteristics prior to sedation

The baseline characteristics of patients experiencing NIV intolerance prior to treatment were presented in Table 3. More than half of the enrolled patients (55.31%) in the present study required NIV due to cardiogenic etiology, and there was no statistically significant difference in the proportion of such patients between the REM and DEX groups (61.8% vs. 49.44%, *p* = 0.097). The median time period from ICU admission to NIV initiation was 58.7 h for all enrolled patients. The difference between REM and DEX groups was not statistically significant (61.0 h, IQR 38–128 h vs. 52.3 h, IQR 36–93 h, *p* = 0.369). The baseline NIS was comparable (3, IQR 3–3 vs. 3, IQR 3–4, *p* = 0.180) between the two groups. When examining the initial NIV settings, including Vt, PS, PEEP, and FiO_2_, it was evident that these differences were also comparable between the two groups. In addition, vital signs, such as the respiratory rate (26 ± 5 breaths/min vs. 28 ± 11 breaths/min, *p* = 0.515), pulse rate, mean arterial blood pressure, SPO_2_, CVP, PaO_2_ (94.05 mmHg, IQR 76.75–140.95 mmHg vs. 95.3 mmHg, IQR 72.70–139.75 mmHg, *p* = 0.824), PaCO_2_ (38.45 mmHg, IQR 34.55–43.30 mmHg vs. 37.85 mmHg, IQR 34.05–44.20 mmHg, *p* = 0.929), laboratory test results, and echocardiography findings, all exhibited comparability between the two groups.

**Table 3:** Baseline characteristics of patients with NIV intolerance prior to treatment

Variables	Total (*n* = 179)	REM group (*n* = 89)	DEX group (*n* = 90)	*p value*
Reasons for NIV				0.097
Cardiogenic, *n* (%)	99 (55.31)	55 (61.80)	44 (49.44)	
Noncardiogenic, *n* (%)	79 (44.13)	34 (38.20)	45 (50.56)	
Duration from ICU admission to NIV (h)	58.7 (37.5, 115.0)	61.0 (38.0, 128.0)	52.3 (36.0, 93.0)	0.369
NIV parameters				
Vt, ml	525 (480, 574)	523 (483, 567)	531 (475, 578)	0.502
PS, cmH_2_O	12 (10, 12)	12 (10, 12)	12 (10, 12)	0.724
PEEP, cmH_2_O	5 (5, 6)	5 (5, 6)	5 (5, 6)	0.695
FiO_2_, %	60 (50, 70)	60 (50, 70)	60 (50, 80)	0.127
VAS, points	3 (2, 3)	3 (2, 3)	3 (2, 3)	0.992
NIS, points	3 (3, 3)	3 (3, 3)	3 (3, 3)	0.180
Vital signs				
Temperature, °C	37 (36.8, 37.7)	37.2 (36.9, 37.8)	37 (36.7, 37.5)	0.024
RR, breaths/min	27 ± 9	26 ± 5	28 ± 11	0.515
HR, bpm	93 (83,105)	93 (83, 104)	91 (81,105)	0.523
SBP, mmHg	125 (114,141)	125 (113,140)	125 (116,145)	0.536
DBP, mmHg	63 ± 11	63 ± 11	63 ± 11	0.842
MAP, mmHg	82 (75, 90)	81(75, 90)	83(76, 90)	0.577
SpO_2_, %	98 (96, 100)	98 (96, 99)	98 (96, 100)	0.425
CVP, mmHg	12(10, 14)	12 (10, 14)	12 (11, 14)	0.831
PaO_2_, mmHg	94.5(74.50,140.85)	94.05(76.75,140.95)	95.3(72.70,139.75)	0.824
PaCO_2_, mmHg	38.3(34.10,43.75)	38.45(34.55,43.30)	37.85(34.05,44.20)	0.929
Laboratory test				
Hb, g/L	90 (82.00, 101.00)	90 (81.50, 99.00)	90 (83.00, 103.00)	0.655
WBC, *10^9^/L	11.47 (8.34, 14.03)	10.84 (8.39, 13.31)	11.94 (8.34, 14.64)	0.302
PLT, *10^9^/L	104 (75, 154)	103.5 (69, 150)	106 (83, 157)	0.180
ALT, U/L	20 (12.00, 36.50)	21.5 (13.50, 46.50)	19 (12.00, 30.50)	0.206
AST, U/L	37 (24.50, 61.00)	42 (25.50, 62.00)	34 (23.50, 59.00)	0.210
TBIL, μmol/L	19.7 (12.80, 30.00)	21.25 (14.30, 30.30)	18.95 (12.20, 29.35)	0.194
DBIL, μmol/L	9.1 (5.70, 16.25)	9.3 (6.20, 16.20)	8.75 (5.34, 16.95)	0.267
CR, μmol/L	106 (81.0, 158.0)	114 (81.0, 158.0)	104 (80.5, 160.0)	0.818
NT-proBNP, pg/ml	2283 (1146, 5201)	2850 (1270, 6074)	1958 (1104, 4581)	0.092
cTnT, ng/ml	0.43 (0.21, 0.90)	0.59 (0.24, 1.00)	0.36 (0.19, 0.83)	0.252
LVEF, %	62 (55, 66)	62 (53, 66)	61 (55, 66)	0.924

Continuous data were presented as mean (SD) or median (IQR). Categorical data were presented as counts (%)

*NIV* noninvasive ventilation, *Vt* tidal volume, *PS* pressure support, *PEEP* positive end expiratory pressure, *FiO*_*2*_ fraction of inspired oxygen, *VAS* visual analogue scale, *NIS* NIV intolerance score, *RR* respiratory rate, *HR* heart rate, *SBP* systolic blood pressure, *DBP* diastolic blood pressure, *MAP* mean arterial pressure, *CVP* central venous pressure, *Hb* hemoglobin, *WBC* white blood cell, *PLT* platelet, *ALT* alanine aminotransferase, *AST* aspartate transaminase, *TBiL* total bilirubin, *DBiL* direct bilirubin, *CR* creatine, *NT-pro BNT* N terminal pro B type natriuretic peptide, *cTnT* cardiac troponin T, *LVEF* left ventricular ejection fraction

### NIV intolerance mitigation

**Fig. 2:**
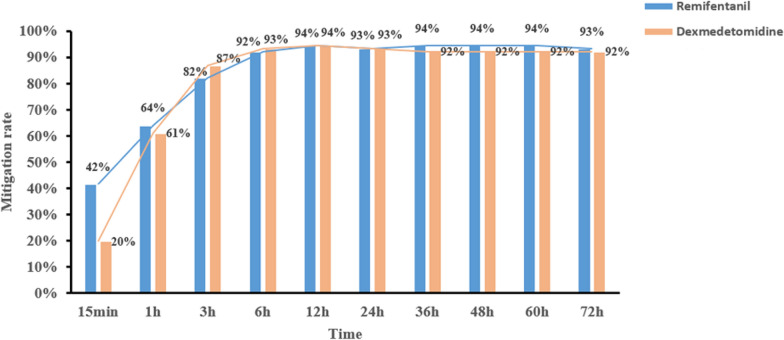
Mitigation rate of NIV intolerance at different timepoints. *NIV* noninvasive ventilation

Throughout the course of the study, the mitigation rates of NIV intolerance exhibited a progressive increase in both groups, which were close to a maximum at 3 h and then leveled off. The REM group showed a significantly higher mitigation rate at the 15-min timepoint (42% vs. 20%,* p* = 0.002). The mitigation rate at 1 h was higher in the REM group but demonstrated no significant difference (64% vs. 61%, *p* = 0.6851). And the mitigation rate of NIV intolerance at different timepoints were shown in Fig. 2.

**Fig. 3:**
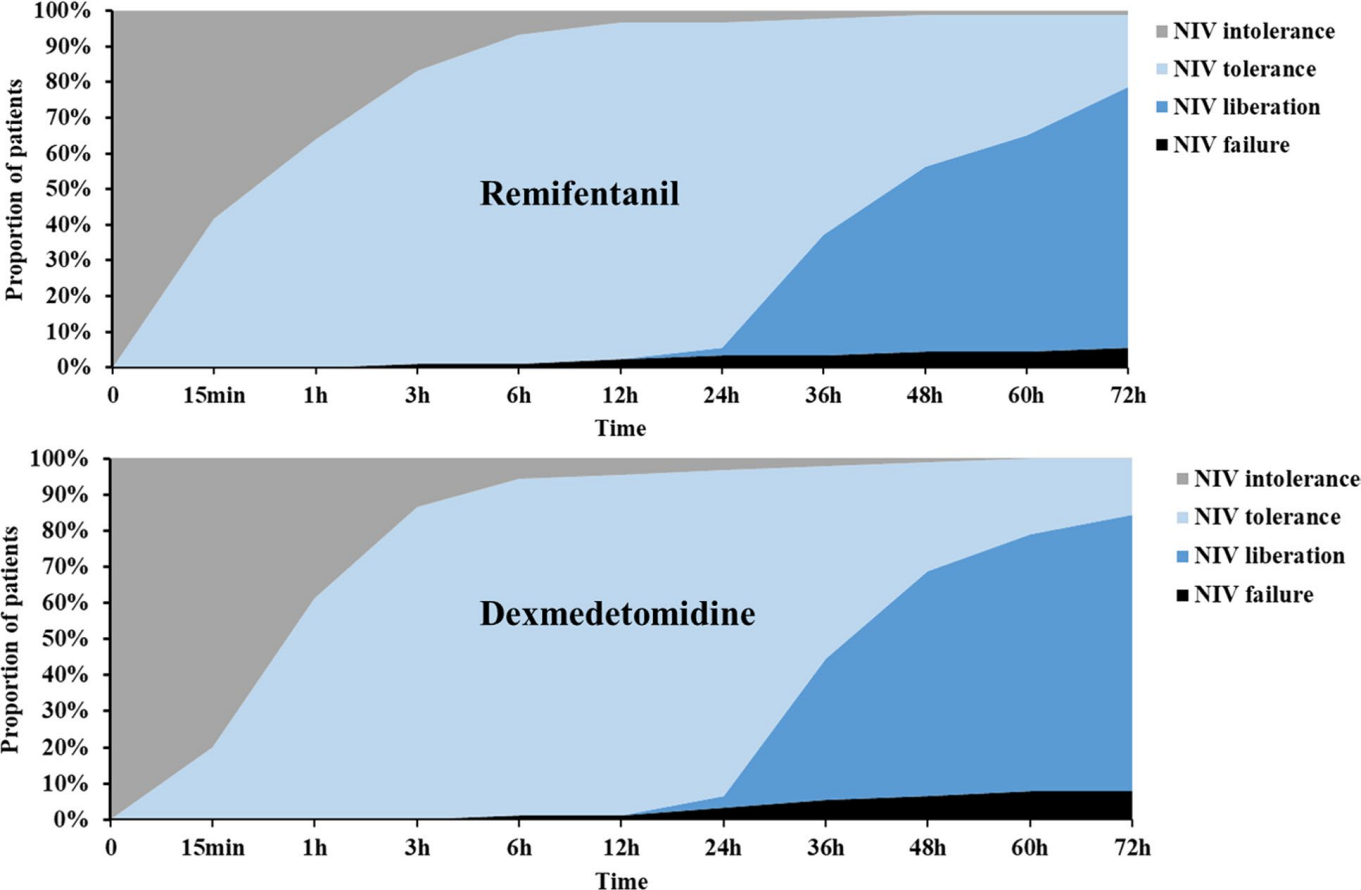
PSA chart for the status of NIV at different timepoints. *PSA* percentage stacked area, *NIV* noninvasive ventilation

The proportion of NIV tolerance and liberation gradually increased after sedation with either REM or DEX. Some patients were unable to achieve relief and required reintubation. In addition, the status of NIV at different timepoints were described in Fig. 3 and Supplemental Table 1.

Taking the cumulative effect of time into account, the difference between the REM and DEX groups was significant and REM outperformed DEX [odds ratio (OR) = 3.31, 95% confidence interval (CI): 1.35–8.12, p = 0.009]. The result of GEE model for NIV intolerance mitigation following sedation was shown in Table 4. In addition, the GEE method was also used to analyze the differences in the efficacy of REM and DEX since the initiation of sedation until 3-, 6-, 12-, 24-, 48-, and 60-h timepoints. The results showed that there were significant differences between the two groups in the mitigation of NIV at all time frames (Supplemental Table 2).

**Table 4:** GEE model for NIV intolerance mitigation following sedation (REM vs. DEX)

Analysis of GEE parameter estimates									
Empirical standard error estimates									
Parameter	Estimate	Standard Error	95% CI		*Z*	*p* value	OR	95% CI	
Intercept	− 1.7645	0.365	− 2.48	− 1.049	− 4.83	< 0.0001			
REM	1.1959	0.4586	0.2972	2.0947	2.61	0.0091	3.3065	1.3461	8.1230
DEX	0	0	0	0					
Time	1.0408	0.1825	0.6831	1.3986	5.7	< 0.0001	2.8315	1.9800	4.0495
Time * REM	− 0.5301	0.2085	− 0.9388	− 0.1213	− 2.54	0.011	0.5886	0.3911	0.8858
Time * DEX	0	0	0	0					

*GEE* generalized estimating equation, *OR* odds ratio, *CI* confidence interval

### Sedative dosages, adverse effects, and patient outcomes

Table 5 provided a summary of dosage of sedatives, adverse effects, and patient outcomes. The highest and lowest DEX doses were 0.5 μg/kg/h and 0.21 μg/kg/h, respectively, whereas the maximum and minimum REM doses were 0.05 μg/kg/min and 0.03 μg/kg/min, respectively. Notably, a total of nine patients (5.03%) encountered AEs during the study, all of which were observed in the DEX group. These AEs included three cases of bradycardia and six cases of severe hypotension. The difference between the two groups was statistically significant (*p* = 0.003). Regarding patient outcomes, including NIV failure (5.6% vs. 7.8%, *p* = 0.564), in-hospital reintubation (7.87% vs. 10%, *p* = 0.617), tracheostomy (1.12% vs. 0%, *p* = 0.313), ICU LOS (7.7 days, IQR 5.8–12 days vs. 7.0 days, IQR 5–10.6 days, *p* = 0.219), and in-hospital mortality (1.12% vs. 2.22%, *p* = 0.567), there were no significant differences between the two groups. In addition, NIV-related parameters, vital signs, and laboratory test results throughout the duration of the study are presented in Supplemental Table 3.

**Table 5:** Medications, adverse effects, and clinical outcomes of patients with NIV intolerance

Variables	Total (*n* = 179)	REM group (*n* = 89)	DEX group (*n* = 90)	*p* value
Duration of NIV, h	47 (35, 72)	49 (36, 72)	45 (31, 71)	0.163
Dosage of sedatives				
Minimum infusion dose, μg/kg/min	–	0.03 (0.02, 0.04)		–
Minimum infusion dose, μg/kg/h	–		0.21 (0.12, 0.39)	–
Maximum infusion dose, μg/kg/min	–	0.05 (0.05, 0.06)		–
Maximum infusion dose, μg/kg/h	–		0.50 (0.45, 0.55)	–
Total daily dose (mg)				
0–24 h	–	2.24 (0.68, 4.00)	0.45 (0.34, 0.68)	–
24–48 h	–	0.58 (0.08, 2.00)	0.10 (0.02, 0.38)	–
48–72 h	–	0.8 (0.00, 2.00)	0.19 (0.00, 0.33)	–
Adverse effects				
Vomiting		0		–
Chest wall rigidity		0		–
Bradycardia		0	3	–
Severe hypotension		0	6	–
NIV failure, *n* (%)	12 (6.70)	5 (5.6)	7 (7.8)	0.564
In-hospital reintubation, *n* (%)	16 (8.94)	7 (7.87)	9 (10.00)	0.617
Tracheostomy, *n *(%)	1 (0.56)	1 (1.12)	0 (0.00)	0.313
ICU LOS, d	7.6 (5.5, 10.7)	7.7 (5.8, 12)	7.0 (5, 10.6)	0.219
ICU events	21 (11.73)	9 (10.11)	12 (13.33)	0.503
BSI, *n* (%)		2 (22.2)	1 (8.3)	
Pneumonia, *n* (%)		6 (66.7)	5 (41.7)	
CRRT, *n* (%)		1 (11.1)	5 (41.7)	
Cerebrovascular events, *n* (%)		0 (0.00)	1 (8.3)	
In-hospital mortality	3 (1.68)	1 (1.12)	2 (2.22)	0.567
Delirium developed within 1 week after 72 h of sedation	2 (1.12)	1 (1.12)	1 (1.11)	0.994

Continuous data were presented as mean (SD) or median (IQR). Categorical data were presented as counts (%)

*NIV* noninvasive ventilation, *LOS* length of stay, *BSI* blood stream infection, *CRRT* continuous renal replacement therapy

## Discussion

The present multicenter, prospective, randomized controlled trial established that REM was as effective as DEX in managing NIV intolerance among cardiac surgery patients with moderate-to-severe symptoms. However, after considering the time factor, the GEE method showed that REM outperformed DEX in improving the mitigation rate of NIV intolerance. In particular, the REM group exhibited a notably higher mitigation rate at the 15-min timepoint and a lower incidence of adverse effects throughout the study. To the best of our knowledge, the present study represents the first multicenter, randomized controlled trial specifically designed to assess and compare the efficacy and safety of REM and DEX in the context of managing NIV intolerance in cardiac surgery patients. Previous studies on this topic were either retrospective [40], single-center and without a sample size calculation [14], or observational and uncontrolled [28, 29], and most of them were conducted in non-cardiac surgery patients. Our research may contribute to the growing body of knowledge surrounding treatment options for this patient population.

A remarkable mitigation of NIV intolerance was observed following sedation in over 90% of the enrolled patients, which was higher than the level noted in the preliminary study [36]. This is likely due to the change in the constitution of enrolled patients. Over 90% of the enrolled patients in the preliminary study had a cardiogenic reason, while over 40% of patients in our study had noncardiogenic reasons. Furthermore, 40% of the enrolled patient in the preliminary study had an NIS of 4, while over 80% of patients in the present study had an NIS of 3.

Rapid onset is an important advantage of REM and a highly attractive feature for patients experiencing NIV intolerance for cardiogenic reasons because increased work of breathing is one of the characteristics of cardiac dysfunction patients [47]. For these patients, intolerance to NIV exacerbated the pre-existing strenuous breathing and tachypnea, and timely mitigation of NIV intolerance was pivotal because failure to act often necessitated intubation. In the present study, an impressive 42% of the REM group’s patients experienced mitigation within just 15 min after initiating sedation. The mitigation rate was notably lower at 20% in the DEX group. This holds significant clinical relevance as evidenced by the fact that the REM group exhibited significantly higher PaO_2_ levels 1 h following sedation initiation compared to the DEX group. Previous studies have demonstrated improvement in oxygenation after 1 h of NIV support as one of the predictors of NIV failure [48, 49]. Furthermore, respiratory rates quickly decreased to an optimal range after sedation initiation in the REM group. This is particularly notable, as prior research has established an association between elevated respiratory rates and increased risk of NIV failure [48, 50].

The chi-squared test failed to show significant differences in mitigation at most of the observed timepoints between the two groups. The efficacy of sedation at different timepoints was the primary outcome in the study. As a result, the dynamic changes in treatment over time need to be considered. This information was part of the longitudinal data for repeated measurement, which had autocorrelation and random error distributed at different levels. For these reasons, the chi-squared method might not be able to reveal the difference. The data collected were the repeated measurement of categorical data, and intra-group non-independence was an inherent problem for the data set. It was thus necessary to use the GEE method in order to solve it. After considering the time factor, the GEE method revealed that the efficacy of REM in the mitigation of NIV intolerance was better than that of DEX, while the onset of sedation was faster.

Presently, persistent NIV intolerance remains a significant contributor to NIV failure, and the reported rates of NIV intolerance were ~ 40–50% [11, 51–55]. The present randomized controlled trial underscored that approximately 25% of patients still experienced NIV intolerance despite comprehensive non-pharmacological interventions. For these patients, sedation—whether with REM or DEX—proved to be highly effective in accordance with the findings of our preliminary study [36]. To date, a consensus on the ideal sedation drug for cardiac surgery patients remains elusive [56–58]. DEX has been consistently ranked among the most thoroughly studied sedatives in the perioperative care over the past decades [23, 59–62]. However, the outcomes of these investigations have yielded inconclusive results and the side effects associated with DEX remain a concern. In the present study, side effects were observed in 10% of the patients enrolled in the DEX group, which included three cases of bradycardia and six cases of severe hypotension. Among these nine patients, six patients’ condition was alleviated after discontinuation of medication use or dose reduction. However, there were still three patients whose condition could not be improved, even after increasing vasoactive drug dosage and reintubation ensued. Indeed, bradycardia and severe hypotension were common DEX side effects, especially for cardiac surgery patients. For example, Alparslan et al. reported 9% of clinically important bradycardia cases and 57% of clinically important hypotension cases after infusion of DEX in the DECADE study [63]. Federico et al. concluded that DEX should be used cautiously in cardiac surgery patients [64]. In the present study, AEs were not observed in the REM group. This might be contributed to the low dose of REM utilized in this study. AEs, such as muscle rigidity, hypotension, and bradycardia, have indeed been reported in cardiac surgery patients after infusion of REM, but the doses in that settings were very high (1–5 μg/kg/min) [65–69]. In this study, the median maximum dose of REM was 0.05 μg/kg/min, which was far from the reported doses resulting in AEs. In a meta-analysis exploring the effect of different doses of REM on postoperative pain, Huang et al. allocated studies with REM infusion less than 0.05 μg/kg/min to the control group [70]. Another study evaluating the efficacy and safety of REM for pain management of Japanese patients in the ICU setting found no AEs leading to discontinuation with a mean infusion rate of 0.046 ± 0.036 μg/kg/min [71]. Besides, the safety of long-term administration of REM in critically ill patients has been studied for up to 5 days [72–75]. As REM has quicker onset and fewer AEs, it might be a better choice for NIV intolerant patients with a cardiogenic reason. However, to verify this, further studies excluding NIV intolerant patients with non-cardiogenic reasons were needed.

This study had several limitations. First, although all enrolled patients underwent cardiac surgery, the causes of NIV intolerance were not exclusively cardiac-related, with approximately 50% attributed to non-cardiogenic factors. Second, the study defined NIV cessation as synonymous with NIV mitigation, which could potentially influence the study’s results. It was difficult to ascertain whether patients were on NIV at all timepoints, especially as their condition improved. In addition, the mitigation rate was close to a maximum according to our preliminary study and tended to be stable after 3 h of treatment [36]. Since almost all patients were still on NIV in the initial 3 h and after 6 h of sedation, patients for whom NIV could be paused were likely not experiencing significant distress, suggesting that they were already NIV-tolerant. Third, most of the enrolled patients had moderate intolerance, which could downgrade the role of sedation. Fourth, we have not collected data on boluses of PCA pump, because in patients undergoing cardiac surgery, the pain was most intense during the first 24 h following the surgery and then declining on subsequent days [76, 77]. In this study, the median duration from ICU admission to initiation of NIV was 58.7 h, and analgesic was routinely provided by a local anesthetic infiltration of ropivacaine, which would significantly decrease the frequency of rescue analgesia [78]. As patients with a VAS score ≥ 4 were excluded, we believed that pain, especially severe pain, was uncommon in this study. Finally, this study did not evaluate the cost of both the study drug and the potential additional healthcare costs associated with AEs.

## Conclusions

In summary, our study demonstrated no significant difference between REM and DEX in the percentage of patients who achieved mitigation among cardiac surgery patients with moderate-to-severe NIV intolerance. However, after considering the time factor, REM was significantly superior to DEX.

## Supplementary Information

Supplementary Material 1

## Data Availability

The data sets used and/or analyzed during the present study are available from the corresponding author on reasonable request.

## Abbreviations

NIV: Noninvasive ventilation
CSICU: Cardiac surgical intensive care unit
DEX: Dexmedetomidine
REM: Remifentanil
NIS: NIV intolerance score
VAS: Visual analogue scale
SBT: Spontaneous breathing trial
PS: Pressure support
PEEP: Positive end-expiratory pressure
Vt: Tidal volume
LOS: Length of stay
RRT: Renal replacement therapy
