# Supplementary material for: Remifentanil vs. dexmedetomidine for cardiac surgery patients with noninvasive ventilation intolerance: a multicenter randomized controlled trial
Source: J Intensive Care. 2024 Sep 18;12:35. doi: 10.1186/s40560-024-00750-2 (PMC11409483; doi:10.1186/s40560-024-00750-2)
Supplement: Supplementary file 1 — Supplementary Material 1 [file 40560_2024_750_MOESM1_ESM.docx]

**Supplemental table 1: NIV status of cardiac surgery patients treated with remifentanil and dexmedetomidine.**

|  |  | 15 min | 1 h | 3 h | 6 h | 12 h | 24 h | 36 h | 48 h | 60 h | 72 h |
| --- | --- | --- | --- | --- | --- | --- | --- | --- | --- | --- | --- |
| REM, n (%) | NIV failure | 0(0.00) | 0(0.00) | 1(1.12) | 1(1.12) | 2(2.25) | 3(3.37) | 3(3.37) | 4(4.49) | 4(4.49) | 5(5.62) |
|  | NIV intolerance | 52(58.43) | 32(35.96) | 15(16.85) | 6(6.74) | 3(3.37) | 3(3.37) | 2(2.25) | 1(1.12) | 1(1.12) | 1(1.12) |
|  | NIV tolerance | 37(41.57) | 57(64.04) | 73(82.02) | 82(92.13) | 84(94.38) | 81(91.01) | 54(60.67) | 38(42.70) | 30(33.71) | 18(20.22) |
|  | NIV liberation | 0(0.00) | 0(0.00) | 0(0.00) | 0(0.00) | 0(0.00) | 2(2.25) | 30(33.71) | 46(51.69) | 54(60.67) | 65(73.03) |
|  | | | | | | | | | | | |
| DEX, n (%) | NIV failure | 0(0.00) | 0(0.00) | 0(0.00) | 1(1.11) | 1(1.11) | 3(3.33) | 5(5.56) | 6(6.67) | 7(7.78) | 7(7.78) |
|  | NIV intolerance | 72(80.00) | 35(38.89) | 12(13.33) | 5(5.56) | 4(4.44) | 3(3.33) | 2(2.22) | 1(1.11) | 0(0.00) | 0(0.00) |
|  | NIV tolerance | 18(20.00) | 55(61.11) | 78(86.67) | 84(93.33) | 85(94.44) | 81(90.00) | 48(53.33) | 27(30.00) | 19(21.11) | 14(15.56) |
|  | NIV liberation | 0(0.00) | 0(0.00) | 0(0.00) | 0(0.00) | 0(0.00) | 3(3.33) | 35(38.89) | 56(62.22) | 64(71.11) | 69(76.67) |
| Overall, n |  | 179 | 179 | 179 | 179 | 179 | 179 | 179 | 179 | 179 | 179 |

**Supplementary table 2: GEE model for early-stage NIV intolerance mitigation following sedation (REM vs. DEX)**

| **Analysis of GEE Parameter Estimates**  **Empirical Standard Error Estimates** | | | | | | | | | |
| --- | --- | --- | --- | --- | --- | --- | --- | --- | --- |
| **Parameter** | **Estimate** | **Standard Error** | **95% CI** | | **Z** | **P-value** | **OR** | **95% CI** | |
| to 3 h | 1.6845 | 0.4854 | 0.7331 | 2.6359 | 3.47 | 0.0005 | 5.3897 | 2.0815 | 13.9558 |
| to 6 h | 1.4500 | 0.4540 | 0.5601 | 2.3399 | 3.19 | 0.0014 | 4.2631 | 1.7508 | 10.3802 |
| to 12 h | 1.3322 | 0.4509 | 0.4484 | 2.2160 | 2.95 | 0.0031 | 3.7893 | 1.5658 | 9.1705 |
| to 24 h | 1.1849 | 0.4390 | 0.3246 | 2.0453 | 2.70 | 0.0069 | 3.2703 | 1.3834 | 7.7314 |
| to 36 h | 0.9334 | 0.4257 | 0.0990 | 1.7678 | 2.19 | 0.0283 | 2.5431 | 1.1041 | 5.8579 |
| to 48 h | 0.8846 | 0.4228 | 0.0558 | 1.7133 | 2.09 | 0.0364 | 2.4220 | 1.0573 | 5.5472 |
| to 60 h | 1.0105 | 0.4427 | 0.1429 | 1.8781 | 2.28 | 0.0224 | 2.7469 | 1.1536 | 6.5410 |

OR, odds ratio; CI, confidence interval.

**Supplemental table 3: NIV-related parameters, vital signs, and laboratory test results over the course of the study**

| **Variables** | **No. at risk** | **Total (n=179)** | **REM (n = 89)** | **DEX (n = 90)** | ***p value*** |
| --- | --- | --- | --- | --- | --- |
| **Vt, mL** |  |  |  |  |  |
| 15 min | 179 | 525(473.00,580.00) | 525(472.00,566.00) | 524(474.00,585.00) | 0.6641 |
| 1 h | 179 | 525(478.00,576.00) | 524(482.00,575.00) | 525(478.00,585.00) | 0.7957 |
| 3 h | 179 | 525(488.00,580.00) | 526(499.00,577.50) | 524(479.00,585.00) | 0.5252 |
| 6 h | 168 | 522(485.50,574.00) | 515(485.00,564.00) | 525(495.00,582.00) | 0.6083 |
| 12 h | 169 | 514(480.00,552.00) | 517(487.00,550.00) | 507(478.00,552.00) | 0.7567 |
| 24 h | 143 | 517(482.00,556.00) | 515(473.00,556.00) | 523(482.50,559.50) | 0.3416 |
| 36 h | 93 | 515(485.00,565.00) | 512(479.00,535.00) | 521(485.00,569.00) | 0.3098 |
| 48 h | 56 | 515(480.50,558.00) | 517.5(492.00,572.50) | 510(475.00,544.50) | 0.4808 |
| 60 h | 50 | 525(485.00,543.00) | 523.5(479.00,568.00) | 526.5(488.00,541.50) | 0.8740 |
| 72 h | 35 | 485(473.00,575.00) | 515(482.00,625.00) | 481(454.50,533.00) | 0.1147 |
| **PS, cmH_2_O** | |  |  |  |  |
| 15 min | 179 | 12(10.00,12.00) | 12(10.00,12.00) | 12(10.00,12.00) | 0.7656 |
| 1 h | 179 | 12(10.00,12.00) | 12(10.00,12.00) | 12(10.00,12.00) | 0.8683 |
| 3 h | 179 | 12(10.00,12.00) | 12(10.00,12.00) | 12(10.00,12.00) | 0.6960 |
| 6 h | 172 | 12(10.00,12.00) | 12(10.00,12.00) | 12(10.00,12.00) | 0.6796 |
| 12 h | 170 | 12(10.00,12.00) | 12(10.00,12.00) | 12(10.00,12.00) | 0.8437 |
| 24 h | 146 | 12(10.00,12.00) | 12(10.00,12.00) | 12(10.00,12.00) | 0.6094 |
| 36 h | 92 | 12(10.00,12.00) | 12(10.00,12.00) | 12(10.00,12.00) | 0.5456 |
| 48 h | 55 | 12(10.00,12.00) | 12(10.00,12.00) | 10(10.00,12.00) | 0.1208 |
| 60 h | 49 | 12(10.00,12.00) | 12(10.00,12.00) | 12(10.00,12.00) | 0.4132 |
| 72 h | 34 | 12(10.00,12.00) | 11(10.00,12.00) | 12(10.00,12.00) | 0.7319 |
| **PEEP, cmH_2_O** | |  |  |  |  |
| 15 min | 179 | 5(5.00,6.00) | 5(5.00,6.00) | 5(5.00,6.00) | 0.5745 |
| 1 h | 179 | 5(5.00,6.00) | 5(5.00,6.00) | 5(5.00,6.00) | 0.5895 |
| 3 h | 179 | 5(5.00,6.00) | 5(5.00,6.00) | 5(5.00,6.00) | 0.3113 |
| 6 h | 172 | 5(5.00,6.00) | 5(5.00,6.00) | 5(5.00,6.00) | 0.5519 |
| 12 h | 170 | 5(5.00,6.00) | 5(5.00,6.00) | 5(5.00,6.00) | 0.8038 |
| 24 h | 146 | 5(5.00,6.00) | 5(5.00,6.00) | 5(5.00,6.00) | 0.3181 |
| 36 h | 92 | 5(5.00,6.00) | 5(5.00,5.00) | 5(5.00,6.00) | 0.1423 |
| 48 h | 55 | 5(5.00,6.00) | 5(5.00,6.00) | 5(5.00,5.50) | 0.2941 |
| 60 h | 49 | 5(5.00,6.00) | 5(5.00,6.00) | 5(5.00,5.00) | 0.2325 |
| 72 h | 34 | 5(5.00,5.00) | 5(5.00,5.00) | 5(5.00,5.00) | 0.9838 |
| **FiO_2_, %** | |  |  |  |  |
| 15 min | 179 | 60(50.00,70.00) | 60(50.00,70.00) | 60(50.00,70.00) | 0.0720 |
| 1 h | 179 | 60(50.00,70.00) | 60(50.00,70.00) | 60(50.00,70.00) | 0.2066 |
| 3 h | 179 | 60(50.00,70.00) | 60(50.00,70.00) | 60(50.00,70.00) | 0.2938 |
| 6 h | 179 | 60(50.00,70.00) | 55(50.00,70.00) | 60(50.00,70.00) | 0.1678 |
| 12 h | 179 | 60(50.00,70.00) | 55(50.00,70.00) | 60(50.00,70.00) | 0.3675 |
| 24 h | 145 | 60(50.00,70.00) | 60(50.00,67.50) | 60(50.00,70.00) | 0.2551 |
| 36 h | 98 | 55(50.00,60.00) | 50(50.00,60.00) | 60(50.00,60.00) | 0.0929 |
| 48 h | 59 | 60(50.00,70.00) | 55(50.00,60.00) | 60(50.00,70.00) | 0.9131 |
| 60 h | 50 | 50(50.00,60.00) | 50(50.00,60.00) | 50(45.00,60.00) | 0.4948 |
| 72 h | 36 | 50(45.00,60.00) | 50(50.00,60.00) | 50(40.00,60.00) | 0.8462 |
| **Temperature, ℃** |  |  |  |  |  |
| 15 min | 179 | 37.1(36.80,37.60) | 37.2(36.90,37.80) | 37(36.70,37.50) | 0.0304 |
| 1 h | 179 | 37.2(36.80,37.70) | 37.3(36.90,37.70) | 37(36.80,37.60) | 0.0414 |
| 3 h | 179 | 37.1(36.80,37.60) | 37.2(36.90,37.70) | 37(36.70,37.50) | 0.0644 |
| 6 h | 179 | 37(36.70,37.60) | 37.1(36.80,37.70) | 37(36.70,37.50) | 0.0907 |
| 12 h | 179 | 37(36.80,37.50) | 37.2(36.90,37.50) | 37(36.70,37.40) | 0.0249 |
| 24 h | 179 | 37(36.80,37.50) | 37(36.80,37.40) | 37(36.70,37.50) | 0.2764 |
| 36 h | 178 | 37(36.80,37.50) | 37(36.80,37.50) | 37(36.70,37.40) | 0.1157 |
| 48 h | 177 | 37(36.70,37.30) | 37(36.80,37.50) | 36.9(36.60,37.20) | 0.0608 |
| 60 h | 176 | 37(36.65,37.20) | 37(36.70,37.20) | 36.9(36.50,37.20) | 0.1303 |
| 72 h | 175 | 36.8(36.60,37.20) | 36.8(36.60,37.20) | 36.8(36.60,37.20) | 0.4747 |
| **RR, breaths/min** | |  |  |  |  |
| 15 min | 179 | 24.15±5.57 | 23.19±4.99 | 25.10±5.97 | 0.0457 |
| 1 h | 179 | 22.70±5.29 | 22.02±5.71 | 23.37±4.77 | 0.0435 |
| 3 h | 179 | 21.47±5.13 | 20.44±5.20 | 22.49±4.87 | 0.0018 |
| 6 h | 179 | 21.03±4.46 | 19.82±4.60 | 22.22±4.00 | 0.0001 |
| 12 h | 179 | 20.41±4.45 | 19.63±4.60 | 21.18±4.18 | 0.0049 |
| 24 h | 179 | 21.25±4.68 | 20.85±4.81 | 21.64±4.54 | 0.2115 |
| 36 h | 178 | 20.93±4.37 | 20.40±4.64 | 21.46±4.04 | 0.0857 |
| 48 h | 177 | 21.35±4.85 | 20.66±5.17 | 22.05±4.43 | 0.0160 |
| 60 h | 176 | 21.33±4.68 | 20.46±4.80 | 22.22±4.42 | 0.0157 |
| 72 h | 174 | 22.13±6.94 | 22.13±8.94 | 22.13±3.94 | 0.2785 |
| **HR, bpm** |  |  |  |  |  |
| 15 min | 179 | 95.11±18.96 | 94.87±17.44 | 95.36±20.46 | 0.8875 |
| 1 h | 179 | 92.82±17.29 | 93.76±15.56 | 91.89±18.89 | 0.1154 |
| 3 h | 179 | 91.36±18.61 | 93.78±17.06 | 88.98±19.83 | 0.0102 |
| 6 h | 179 | 89.65±17.62 | 94.24±16.88 | 85.12±17.25 | 0.0004 |
| 12 h | 179 | 90.06±18.30 | 94.73±17.30 | 85.43±18.18 | 0.0004 |
| 24 h | 179 | 92.12±17.01 | 94.81±17.13 | 89.47±16.56 | 0.0449 |
| 36 h | 178 | 90.54±16.19 | 91.79±16.47 | 89.29±15.90 | 0.4244 |
| 48 h | 177 | 91.67±15.96 | 92.09±16.87 | 91.25±15.07 | 0.8407 |
| 60 h | 176 | 89.80±15.68 | 90.53±16.62 | 89.05±14.71 | 0.7899 |
| 72 h | 174 | 90.32±13.85 | 90.36±13.15 | 90.28±14.62 | 0.6535 |
| **SBP, mmHg** |  |  |  |  |  |
| 15 min | 179 | 127.82±19.66 | 126.30±17.58 | 129.31±21.51 | 0.3543 |
| 1 h | 179 | 124.02±19.45 | 124.56±17.54 | 123.49±21.26 | 0.6682 |
| 3 h | 179 | 122.49±19.57 | 124.38±19.50 | 120.62±19.57 | 0.1565 |
| 6 h | 179 | 120.90±18.63 | 122.49±19.29 | 119.32±17.92 | 0.1678 |
| 12 h | 179 | 122.39±18.97 | 122.99±18.25 | 121.80±19.75 | 0.4385 |
| 24 h | 179 | 126.21±19.21 | 123.94±18.39 | 128.44±19.83 | 0.1523 |
| 36 h | 178 | 125.31±17.96 | 124.21±17.92 | 126.40±18.03 | 0.4467 |
| 48 h | 177 | 126.66±18.50 | 122.67±16.37 | 130.69±19.71 | 0.0178 |
| 60 h | 176 | 126.32±16.73 | 124.10±15.77 | 128.59±17.45 | 0.2352 |
| 72 h | 173 | 126.19±16.52 | 123.91±16.06 | 128.61±16.76 | 0.0755 |
| **DBP, mmHg** |  |  |  |  |  |
| 15 min | 179 | 62.10±10.92 | 61.85±11.34 | 62.34±10.56 | 0.7312 |
| 1 h | 179 | 61.00±10.41 | 61.91±10.24 | 60.10±10.55 | 0.2566 |
| 3 h | 179 | 60.59±10.99 | 62.20±12.15 | 58.99±9.52 | 0.1113 |
| 6 h | 179 | 61.04±10.62 | 62.37±10.74 | 59.72±10.39 | 0.0636 |
| 12 h | 179 | 61.55±9.30 | 62.53±9.22 | 60.58±9.34 | 0.1592 |
| 24 h | 179 | 63.42±11.66 | 64.19±12.41 | 62.67±10.89 | 0.5894 |
| 36 h | 178 | 62.62±10.91 | 63.15±10.02 | 62.10±11.77 | 0.3257 |
| 48 h | 177 | 62.77±9.36 | 62.00±9.44 | 63.55±9.27 | 0.1369 |
| 60 h | 176 | 63.73±9.49 | 63.38±9.12 | 64.09±9.90 | 0.479 |
| 72 h | 173 | 63.90±9.67 | 63.69±9.21 | 64.13±10.19 | 0.7255 |
| **MAP, mmHg** |  |  |  |  |  |
| 15 min | 179 | 82.17±10.94 | 82.18±10.86 | 82.17±11.07 | 0.7519 |
| 1 h | 179 | 80.08±11.39 | 80.93±10.73 | 79.24±12.01 | 0.1920 |
| 3 h | 179 | 79.81±11.65 | 82.11±12.27 | 77.53±10.59 | 0.0255 |
| 6 h | 179 | 80.12±11.52 | 81.75±11.80 | 78.51±11.07 | 0.0348 |
| 12 h | 179 | 80.32±10.39 | 81.88±10.20 | 78.79±10.42 | 0.1109 |
| 24 h | 179 | 82.57±10.93 | 82.93±10.64 | 82.20±11.26 | 0.6587 |
| 36 h | 178 | 81.21±9.99 | 81.35±10.72 | 81.07±9.26 | 0.9327 |
| 48 h | 177 | 82.64±10.36 | 80.58±9.52 | 84.72±10.81 | 0.0058 |
| 60 h | 176 | 82.53±11.32 | 82.49±9.75 | 82.57±12.79 | 0.6398 |
| 72 h | 173 | 83.04±10.04 | 82.62±9.55 | 83.48±10.57 | 0.6993 |
| **CVP, cmH_2_O** | |  |  |  |  |
| 15 min | 179 | 12(10.00,14.00) | 12(10.00,14.00) | 12(11.00,14.00) | 0.5600 |
| 1 h | 179 | 12(11.00,13.00) | 12(10.00,13.00) | 12(11.00,13.00) | 0.7355 |
| 3 h | 179 | 12(10.00,13.00) | 12(10.00,14.00) | 12(11.00,13.00) | 0.3848 |
| 6 h | 178 | 12(10.00,13.00) | 12(11.00,14.00) | 12(10.00,13.00) | 0.1103 |
| 12 h | 179 | 12(11.00,13.00) | 12(11.00,13.00) | 12(10.00,13.00) | 0.4024 |
| 24 h | 179 | 11(10.00,13.00) | 12(10.00,13.00) | 11(10.00,13.00) | 0.2594 |
| 36 h | 178 | 11(10.00,13.00) | 12(10.00,13.00) | 11(10.00,13.00) | 0.1019 |
| 48 h | 173 | 11(10.00,13.00) | 11(10.00,13.00) | 11(10.00,12.00) | 0.2139 |
| 60 h | 171 | 11(10.00,13.00) | 11(10.00,13.00) | 11(10.00,13.00) | 0.7046 |
| 72 h | 167 | 11(9.00,12.00) | 11(9.00,12.00) | 10(9.00,12.00) | 0.1128 |
| **PaO_2_, mmHg** |  |  |  |  |  |
| 15 min | 58 | 91.2(74.40,140.50) | 100(85.00,147.50) | 83(71.00,120.70) | 0.0737 |
| 1 h | 50 | 109(77.40,143.00) | 121(99.10,181.50) | 89.5(70.00,124.00) | 0.0178 |
| 3 h | 92 | 105(77.90,151.80) | 107.65(82.20,149.05) | 104.95(75.95,159.00) | 0.9564 |
| 6 h | 76 | 116.6(85.45,150.85) | 115(85.40,152.30) | 119(85.60,149.40) | 0.7030 |
| 12 h | 110 | 116.6(86.50,156.00) | 120.6(83.10,156.20) | 115.6(88.90,154.80) | 0.9547 |
| 24 h | 149 | 120.9(86.70,169.20) | 126.5(88.00,174.50) | 118.4(83.30,166.10) | 0.2843 |
| 36 h | 131 | 121.3(93.20,160.00) | 119.8(100.00,166.20) | 125.45(88.50,151.60) | 0.4855 |
| 48 h | 136 | 129.3(89.90,169.25) | 132.6(99.90,167.60) | 121.6(88.30,172.80) | 0.3174 |
| 60 h | 117 | 109.6(88.40,147.20) | 120.55(89.90,151.80) | 100.9(82.10,136.60) | 0.1113 |
| 72 h | 113 | 123.5(88.90,166.20) | 125.1(90.20,173.20) | 117.5(84.40,160.45) | 0.6459 |
| **PaCO_2_, mmHg** | |  |  |  |  |
| 15 min | 58 | 39.4(34.50,43.40) | 39.3(35.80,42.50) | 39.8(34.10,43.90) | 0.8440 |
| 1h | 50 | 38.6(33.60,43.00) | 39(35.00,44.00) | 36.7(31.90,43.00) | 0.3335 |
| 3 h | 92 | 37.4(34.40,42.60) | 38.95(34.90,45.20) | 37(34.10,42.00) | 0.2079 |
| 6 h | 76 | 38.5(33.70,44.70) | 39(33.20,47.00) | 38.1(33.70,42.70) | 0.5299 |
| 12 h | 110 | 39.7(35.50,44.40) | 40.85(35.80,46.50) | 38.4(35.20,44.00) | 0.2916 |
| 24 h | 149 | 39.1(34.80,42.60) | 39.3(35.20,43.40) | 37.85(34.30,42.00) | 0.4593 |
| 36 h | 131 | 37.9(34.20,42.00) | 38.1(34.30,41.50) | 37.5(34.20,42.10) | 0.9703 |
| 48 h | 136 | 37.9(34.00,42.00) | 37.7(34.10,42.00) | 38.05(33.95,41.85) | 0.9515 |
| 60 h | 117 | 38.5(35.00,43.10) | 39.4(35.45,43.30) | 36.4(34.10,41.30) | 0.2147 |
| 72 h | 113 | 37(33.15,41.85) | 39(34.80,41.85) | 35.9(31.90,41.90) | 0.1363 |
| **SPO_2_, %** |  |  |  |  |  |
| 15 min | 179 | 98(96.00,100.00) | 98(96.00,100.00) | 98(96.00,100.00) | 0.6576 |
| 1 h | 179 | 99(97.00,100.00) | 99(97.00,100.00) | 98(97.00,100.00) | 0.9110 |
| 3 h | 179 | 99(97.00,100.00) | 99(97.00,100.00) | 99(98.00,100.00) | 0.1128 |
| 6 h | 179 | 99(97.00,100.00) | 99(97.00,100.00) | 99(98.00,100.00) | 0.8544 |
| 12 h | 179 | 99(98.00,100.00) | 99(98.00,100.00) | 99(98.00,100.00) | 0.9297 |
| 24 h | 179 | 99(98.00,100.00) | 99(98.00,100.00) | 99(98.00,100.00) | 0.9570 |
| 36 h | 176 | 99(98.00,100.00) | 99(98.00,100.00) | 99(98.00,100.00) | 0.1274 |
| 48 h | 175 | 99(98.00,100.00) | 99(98.00,100.00) | 99(98.00,100.00) | 0.6127 |
| 60 h | 174 | 99(98.00,100.00) | 100(98.00,100.00) | 99(97.00,100.00) | 0.0384 |
| 72 h | 169 | 100(98.00,100.00) | 100(98.00,100.00) | 99(97.00,100.00) | 0.0708 |
| **VAS, points** |  |  |  |  |  |
| 15 min | 179 | 2(1.00,3.00) | 2(1.00,3.00) | 2(2.00,3.00) | 0.2356 |
| 1 h | 179 | 2(1.00,3.00) | 2(1.00,3.00) | 2(1.00,3.00) | 0.8234 |
| 3 h | 179 | 2(1.00,2.00) | 2(1.00,2.00) | 2(1.00,2.00) | 0.7812 |
| 6 h | 178 | 1(1.00,2.00) | 2(1.00,2.00) | 1(1.00,2.00) | 0.1472 |
| 12 h | 179 | 1(1.00,2.00) | 1(1.00,2.00) | 1(1.00,2.00) | 0.0966 |
| 24 h | 177 | 1(1.00,2.00) | 1(1.00,2.00) | 1(1.00,2.00) | 0.4775 |
| 36 h | 178 | 1(1.00,1.00) | 1(1.00,1.00) | 1(1.00,1.00) | 0.7070 |
| 48 h | 178 | 1(1.00,1.00) | 1(1.00,1.00) | 1(1.00,1.00) | 0.5963 |
| 60 h | 176 | 1(1.00,1.00) | 1(1.00,1.00) | 1(1.00,1.00) | 0.0174 |
| 72 h | 176 | 1(1.00,1.00) | 1(1.00,1.00) | 1(1.00,1.00) | 0.1031 |
| **NIS, points** |  |  |  |  |  |
| 15 min | 179 | 3(2.00,3.00) | 3(2,3) | 3(3,3) | 0.0012 |
| 1 h | 179 | 2(2.00,3.00) | 2(2,3) | 2(2,3) | 0.2515 |
| 3 h | 178 | 2(1.00,2.00) | 2(1,2) | 2(2,2) | 0.0285 |
| 6 h | 177 | 2(1.00,2.00) | 2(1,2) | 2(1,2) | 0.0778 |
| 12 h | 175 | 1(1.00,2.00) | 1(1,2) | 1(1,2) | 0.4702 |
| 24 h | 162 | 1(1.00,2.00) | 1(1,2) | 1(1,2) | 0.4886 |
| 36 h | 94 | 1(1.00,2.00) | 1(1,2) | 1(1,2) | 0.8575 |
| 48 h | 54 | 1(1.00,2.00) | 1(1,2) | 1(1,2) | 0.9916 |
| 60 h | 43 | 1(1.00,2.00) | 1(1,2) | 1(1,1) | 0.1354 |
| 72 h | 29 | 1(1.00,1.00) | 1(1,1) | 1(1,1) | 0.2958 |
| **Dosage of REM, μg/kg/min** |  |  |  |  |  |
| 15 min | 179 |  | 0.05(0.05,0.06) |  | / |
| 1 h | 179 |  | 0.05(0.05,0.06) |  | / |
| 3 h | 178 |  | 0.05(0.04,0.06) |  | / |
| 6 h | 169 |  | 0.04(0.03,0.05) |  | / |
| 12 h | 163 |  | 0.04(0.03,0.05) |  | / |
| 24 h | 122 |  | 0.04(0.02,0.05) |  | / |
| 36 h | 72 |  | 0.04(0.02,0.05) |  | / |
| 48 h | 35 |  | 0.04(0.03,0.06) |  | / |
| 60 h | 29 |  | 0.04(0.02,0.05) |  | / |
| 72 h | 20 |  | 0.03(0.02,0.05) |  | / |
| **Dosage of DEX, μg/kg/h** |  |  |  |  |  |
| 15 min | 179 |  |  | 0.49(0.43,0.51) | / |
| 1 h | 179 |  |  | 0.46(0.34,0.53) | / |
| 3 h | 178 |  |  | 0.43(0.28,0.50) | / |
| 6 h | 169 |  |  | 0.38(0.24,0.49) | / |
| 12 h | 163 |  |  | 0.32(0.20,0.44) | / |
| 24 h | 122 |  |  | 0.24(0.15,0.40) | / |
| 36 h | 72 |  |  | 0.18(0.12,0.28) | / |
| 48 h | 35 |  |  | 0.24(0.11,0.38) | / |
| 60 h | 29 |  |  | 0.18(0.11,0.34) | / |
| 72 h | 20 |  |  | 0.27(0.17,0.45) | / |
| **Delirium, %** |  |  |  |  |  |
| 1 h | 179 | 1(0.56) | 0(0.00) | 1(1.11) | 1.0000 |
| 3 h | 179 | 0(0.00) | 0(0.00) | 0(0.00) | 0.3187 |
| 6 h | 179 | 0(0.00) | 0(0.00) | 0(0.00) | 0.3187 |
| 12 h | 179 | 0(0.00) | 0(0.00) | 0(0.00) | 0.3187 |
| 24 h | 179 | 1(0.56) | 1(1.14) | 0(0.00) | 0.4972 |
| 36 h | 179 | 3(1.68) | 1(1.12) | 2(2.22) | 1.0000 |
| 48 h | 179 | 2(1.12) | 1(1.12) | 1(1.11) | 1.0000 |
| 60 h | 179 | 1(0.56) | 1(1.14) | 0(0.00) | 1.0000 |
| 72 h | 179 | 2(1.12) | 2(2.27) | 0(0.00) | 0.4971 |
| **Hb, g/L** |  |  |  |  |  |
| 0-24 h | 179 | 90(81.00,101.00) | 91(81.00,102.00) | 89.5(80.00,99.00) | 0.6958 |
| 24-48 h | 174 | 92.5(83.00,103.00) | 92(83.00,102.00) | 93(81.00,107.00) | 0.8190 |
| 48-72 h | 174 | 92.5(85.00,105.00) | 89(82.50,101.50) | 95.5(86.00,107.00) | 0.0734 |
| **WBC, 10^^^9/L** |  |  |  |  |  |
| 0-24 h | 179 | 10.69(7.71,14.25) | 11.02(7.76,14.14) | 10.36(7.71,14.30) | 0.6218 |
| 24-48 h | 174 | 10.08(7.34,13.10) | 10.4(7.83,12.79) | 9.41(7.28,13.55) | 0.4374 |
| 48-72 h | 174 | 9.68(7.43,12.90) | 9.71(7.46,12.25) | 9.63(7.26,13.64) | 0.6724 |
| **PLT, 10^^^9/L** |  |  |  |  |  |
| 0-24 h | 179 | 110(84.00,169.00) | 109(85.00,156.00) | 111(84.00,172.00) | 0.3551 |
| 24-48 h | 174 | 136.5(101.00,196.00) | 133(99.00,190.00) | 137(102.00,204.00) | 0.5146 |
| 48-72 h | 173 | 162(114.00,219.00) | 151(107.00,207.50) | 171(120.00,224.00) | 0.1766 |
| **ALT, U/L** |  |  |  |  |  |
| 0-24 h | 172 | 21(12.50,45.00) | 25(12.00,50.00) | 18(13.00,43.00) | 0.2954 |
| 24-48 h | 166 | 23(13.00,44.00) | 25(14.00,49.00) | 22(12.00,43.00) | 0.3275 |
| 48-72 h | 168 | 26(14.00,48.50) | 25.5(14.00,47.50) | 27(13.50,49.00) | 0.7874 |
| **AST, U/L** |  |  |  |  |  |
| 0-24 h | 172 | 33(22.00,57.00) | 36(24.00,60.00) | 32(20.00,52.00) | 0.3128 |
| 24-48 h | 166 | 29.5(21.00,49.00) | 31(22.00,47.00) | 28(20.00,50.00) | 0.2775 |
| 48-72 h | 167 | 30(19.00,43.00) | 29(19.00,43.00) | 30(19.00,45.00) | 0.9617 |
| **TBIL, μmol/L** |  |  |  |  |  |
| 0-24 h | 172 | 19.6(13.50,31.05) | 18.5(14.60,31.10) | 20.7(11.70,31.00) | 0.4764 |
| 24-48 h | 166 | 19.85(13.10,31.30) | 20.8(14.70,35.00) | 18.2(11.60,29.10) | 0.1356 |
| 48-72 h | 168 | 19.05(14.75,29.00) | 20(15.20,31.10) | 18.45(13.80,27.00) | 0.3325 |
| **DBIL, μmol/L** |  |  |  |  |  |
| 0-24 h | 172 | 9.1(5.40,15.50) | 9.2(5.90,17.10) | 9(4.80,15.10) | 0.2886 |
| 24-48 h | 166 | 9.25(5.00,14.80) | 10.7(5.70,18.30) | 8.6(4.60,13.10) | 0.1229 |
| 48-72 h | 169 | 9.1(5.80,15.50) | 9.9(6.30,18.80) | 8.35(5.25,14.25) | 0.1203 |
| **CR, μmol/L** |  |  |  |  |  |
| 0-24 h | 173 | 103(76.00,158.00) | 102.5(76.50,148.00) | 103(76.00,159.00) | 0.7190 |
| 24-48 h | 164 | 92(69.50,143.00) | 92(71.00,144.00) | 92(69.00,142.00) | 0.8461 |
| 48-72 h | 170 | 82.5(64.00,126.00) | 82.5(68.00,132.00) | 82.5(62.00,119.00) | 0.6603 |
| **NT-proBNP, pg/mL** |  |  |  |  |  |
| 0-24 h | 176 | 2918.5(1424.50,6252.00) | 3629(1765.50,7255.50) | 1956(1216.00,5686.50) | 0.0161 |
| 24-48 h | 170 | 2838.5(1370.00,6255.00) | 3838(1722.00,7111.00) | 2399(1222.00,4734.00) | 0.0279 |
| 48-72 h | 171 | 2571(1189.00,5489.00) | 2725.5(1636.00,7255.00) | 2133(1013.00,4715.00) | 0.0270 |
| **cTnT, ng/mL** |  |  |  |  |  |
| 0-24 h | 177 | 0.35(0.16,0.79) | 0.43(0.17,0.79) | 0.3(0.16,0.80) | 0.6503 |
| 24-48 h | 171 | 0.3(0.13,0.72) | 0.33(0.14,0.77) | 0.25(0.13,0.70) | 0.3312 |
| 48-72 h | 169 | 0.23(0.10,0.58) | 0.27(0.11,0.62) | 0.21(0.09,0.56) | 0.4074 |

Continuous data are presented as mean (SD) or median (IQR). Categorical data are presented as counts (%).Vt, tidal volume; PS, pressure support; PEEP, positive end expiratory pressure; FiO_2_, fraction of inspired oxygen; VAS, visual analogue scale; NIS, NIV intolerance score; RR, respiratory rate; HR, heart rate; SBP, systolic blood pressure; DBP, diastolic blood pressure; MAP, mean arterial pressure; CVP, central venous pressure; Hb, hemoglobin; WBC, white blood cell; PLT, platelet; ALT, alanine aminotransferase; AST, aspartate transaminase; TBiL, total bilirubin; DBiL, direct bilirubin; CR, creatine; NT-pro BNT, N-terminal pro-B-type natriuretic peptide; cTnT, cardiac troponin T.
